# Supplementary material for: Methanolic Extracts of D. viscosa Specifically Affect the Cytoskeleton and Exert an Antiproliferative Effect on Human Colorectal Cancer Cell Lines, According to Their Proliferation Rate
Source: Int J Mol Sci. 2023 Oct 5;24(19):14920. doi: 10.3390/ijms241914920 (PMC10573359; doi:10.3390/ijms241914920)
Supplement: Supplementary file 1 [file ijms-24-14920-s001.zip › ijms-2625015-supplementary.pdf]

Methanolic extracts of D. viscosa specifically affects cytoskeleton and exerts its antiproliferative effect on human colorectal cancer cell lines, accordingly to their proliferation rate

Chiara Anglana [1]†, Makarena Rojas [1]†, Chiara Roberta Girelli [1], Fabrizio Barozzi [1], Josefa Quiroz-Troncoso [2], Nicolás Alegría-Aravena [2, 3], Anna Montefusco [1], Miriana Durante [4], Francesco Paolo Fanizzi [1], Carmen Ramírez-Castillejo [2] and Gian-Pietro Di Sansebastiano [1]

Supplementary Table S1. *p*-value of the comparison between the treated cells lines shown in **Figure 6**

Treatment	Concentration (µg/mL)	Comparison	*p*-value
**Hexanic extract**	60	DLD-1/HT-29	<0.05
**Hexanic extract**	60	HT-29/SW480	<0.05
**Hexanic extract**	90	SW480/SW620	<0.05
**Hexanic extract**	180	DLD-1/SW620	<0.01
**Methanolic extract**	50	SW480/SW620	<0.0001
**Methanolic extract**	100	SW480/SW620	<0.05
**Methanolic extract**	150	HT-29/SW480	<0.01
**Methanolic extract**	150	SW480/SW620	<0.01
**Aqueous extract**	400	SW480/SW620	<0.01
**Aqueous extract**	500	DLD-1/SW620	<0.0001
**Aqueous extract**	500	HT-29/SW620	<0.0001
**Aqueous extract**	500	SW480/SW620	<0.0001
**Aqueous extract**	600	SW480/SW620	<0.01
**Aqueous extract**	1000	DLD-1/SW480	<0.01
**Aqueous extract**	1000	DLD-1/SW620	<0.0001
**Aqueous extract**	1000	HT-29/SW480	<0.0001
**Aqueous extract**	1000	HT-29/SW620	<0.0001
**Aqueous extract**	1000	SW480/SW620	<0.01
**Aqueous extract**	1500	DLD-1/SW480	<0.01
**Aqueous extract**	1500	DLD-1/SW620	<0.001
**Aqueous extract**	1500	HT-29/SW480	<0.0001
**Aqueous extract**	1500	HT-29/SW620	<0.0001
**Aqueous extract**	2000	DLD-1/SW480	<0.05
**Aqueous extract**	2000	DLD-1/SW620	<0.01
**Aqueous extract**	2000	HT-29/SW480	<0.01
**Aqueous extract**	2000	HT-29/SW620	<0.01
**Aqueous extract**	2500	DLD-1/SW620	<0.05
**Aqueous extract**	3000	DLD-1/SW480	<0.0001
**Aqueous extract**	3000	DLD-1/SW620	<0.0001
**Aqueous extract**	3000	HT-29/SW480	<0.01

Supplementary Table S2. IC50 values of each cell line exposed under different treatments.

Treatment	Cell line	IC50 Concentration (µg/mL)	Statistical error
**Aqueous extract**	DLD-1	1923	±139
**Aqueous extract**	HT-29	1865	±58
**Aqueous extract**	SW480	1031	±25
**Aqueous extract**	SW620	405	±74
**Hexanic extract**	DLD-1	159	±13
**Hexanic extract**	HT-29	126	±17
**Hexanic extract**	SW480	128	±11
**Hexanic extract**	SW620	87	±13
**Methanolic extract**	DLD-1	142	±2
**Methanolic extract**	HT-29	15	±2
**Methanolic extract**	SW480	122	±2
**Methanolic extract**	SW620	66	±2
